# Trisomy 12 compromises the mesendodermal differentiation propensity of human pluripotent stem cells

**DOI:** 10.1007/s11626-023-00824-9

**Published:** 2024-01-02

**Authors:** Kana Yanagihara, Yohei Hayashi, Yujung Liu, Tomoko Yamaguchi, Yasuko Hemmi, Minako Kokunugi, Kozue Uchio Yamada, Ken Fukumoto, Mika Suga, Satoshi Terada, Hiroki Nikawa, Kenji Kawabata, Miho Furue

**Affiliations:** 1 Laboratory of Stem Cell Cultures, National Institutes of Biomedical Innovation, Health, and Nutrition, 7-6-8, Saito-Asagi, Osaka, Ibaraki 567-0085 Japan; 2 iPS Cell Advanced Characterization and Development Team, RIKEN Bioresource Research Center, 3-1-1 Koyadai, Tsukuba, Ibaraki 305-0074 Japan; 3 Laboratory of Cell Model for Drug Discovery, National Institutes of Biomedical Innovation, Health and Nutrition, 7-6-8, Saito-Asagi, Osaka, Ibaraki 567-0085 Japan; 4 Department of Oral Biology & Engineering Integrated Health Sciences, Institute of Biomedical and Health Sciences, Hiroshima University, Hiroshima, Japan; 5 Laboratory of Animal Models for Human Diseases, National Institutes of Biomedical Innovation, Health, and Nutrition, 7-6-8, Saito-Asagi, Osaka, Ibaraki 567-0085 Japan; 6 Department of Applied Chemistry and Biotechnology, University of Fukui, Fukui City, 3-9-1 Bunkyo, Fukui, 910-8507 Japan; 7 Present Address: Cel-MiM, Ltd., Tokyo, Japan

**Keywords:** Trisomy 12, Pluripotent stem cells, Mesendodermal differentiation, BMP4

## Abstract

Trisomy 12 is one of the most frequent chromosomal abnormalities in cultured human pluripotent stem cells (hPSCs). Although potential oncogenic properties and augmented cell cycle caused by trisomy 12 have been reported, the consequences of trisomy 12 in terms of cell differentiation, which is the basis for regenerative medicine, drug development, and developmental biology studies, have not yet been investigated. Here, we report that trisomy 12 compromises the mesendodermal differentiation of hPSCs. We identified sublines of hPSCs carrying trisomy 12 after their prolonged culture. Transcriptome analysis revealed that these hPSC sublines carried abnormal gene expression patterns in specific signaling pathways in addition to cancer-related cell cycle pathways. These hPSC sublines showed a lower propensity for mesendodermal differentiation in embryoid bodies cultured in a serum-free medium. BMP4-induced exit from the self-renewal state was impaired in the trisomy 12 hPSC sublines, with less upregulation of key transcription factor gene expression. As a consequence, the differentiation efficiency of hematopoietic and hepatic lineages was also impaired in the trisomy 12 hPSC sublines. We reveal that trisomy 12 disrupts the genome-wide expression patterns that are required for proper mesendodermal differentiation.

**Supplementary Information:**

The online version contains supplementary material available at 10.1007/s11626-023-00824-9.

## Introduction

Human pluripotent stem cells (hPSCs), including human embryonic stem cells (hESCs) (Thomson *et al*. 1998) and human induced pluripotent stem cells (hiPSCs) (Takahashi *et al*. 2007; Yu *et al*. 2007), can replicate indefinitely and differentiate into most cell types in the body (Murry and Keller 2008; Barbaric *et al*. 2010; Engle and Puppala 2013). Thus, hPSCs are anticipated to be promising cell resources for basic biological research, cell-based medicine, and drug development. Maintaining the quality of hPSCs is critical for obtaining reproducible and reliable data in these research areas. In particular, the genomic integrity of hPSCs should be maintained. During prolonged culture, however, hPSCs often acquire chromosomal abnormalities (Draper *et al*. 2004, Enver *et al*. 2005, Baker *et al*. 2007, Olariu et al. 2010, International Stem Cell Initiative *et al*. 2011, Na *et al*. 2014, Barbaric *et al*. 2014). Previous studies, including large-scale and international projects, have revealed that trisomy 12 is one of the most prevalent chromosomal abnormalities (Draper *et al*. 2004, International Stem Cell Initiative *et al*. 2011, Taapken *et al*. 2011).

In view of disease modeling and drug development as well as basic research in developmental biology, the consequences of trisomy 12 in terms of hPSC differentiation should be elucidated (Hayashi *et al*. 2020). In this study, we examined the effects of trisomy 12 on the differentiation potentials and propensities of three hPSC sublines carrying trisomy 12 from established hPSC lines by means of embryoid formation and direct differentiation methods. While the trisomy 12 hPSC sublines maintained pluripotency overall, they showed less propensity toward mesendodermal differentiation, as revealed by embryoid body formation or direct differentiation analysis in a serum-free medium. Combined with analyses of global gene expression profiles, these data demonstrate the critical roles of chromosome 12 in modulating the expression patterns and cell signaling pathways required for proper mesendodermal differentiation.

## Materials and methods

### Ethics statement

hESCs were used at the National Institutes of Biomedical Innovation, Health and Nutrition (NIBIOHN) according to the “Guidelines for Utilization of Human Embryonic Stem Cells” of the Ministry of Education, Culture, Sports, Science and Technology of Japan after approval by the Institutional Ethical Review Board of the NIBIOHN. Experiments using human iPSCs were conducted with the approval of NIBIOHN and RIKEN BioResource Research Center.

### Cell culture

A human ESC line H9 (WA09) and a hiPSC line 19–9-7 T were provided by WISC Bank, WiCell Research Institute, Madison, WI (Thomson *et al*. 1998; Amit *et al*. 2000), and cultured only in the National Institute of Biomedical Innovation. The human iPSC lines, namely, parental and abnormal 201B7, were provided by Dr. Shinya Yamanaka, Kyoto University (Takahashi *et al*. 2007). Human ES/iPSCs with normal or abnormal chromosomes were routinely maintained on bovine fibronectin (2 μg/cm^2^) in a serum-free hESF-FX medium. The hESF-FX medium consisted of mESF basal medium (WAKO, Osaka, Japan) supplemented with three factors (3F: 10 µM 2-mercaptoethanol (Sigma-Aldrich, St. Louis, MO), 20 nM sodium selenite (Sigma-Aldrich), 10 µM 2-aminoethanol (Sigma-Aldrich)); 1 mg/ml oleic acid (Sigma-Aldrich)-conjugated recombinant human serum albumin (rHSA, Sigma-Aldrich); 10 µg/ml human insulin (Sigma-Aldrich); 5 µg/ml apo-transferrin (Sigma-Aldrich); 100 µg/ml L-Ascorbic acid 2-phosphate (WAKO); 5 ng/ml human recombinant FGF-2 (Katayama Kagaku Kogyo LTD., Osaka, Japan); and 2 ng/ml human Activin A (R&D systems, Minneapolis, MN) for seeding and without Activin A for culture maintenance (Furue *et al*. 2008; Kinehara *et al*. 2013; Yanagihara *et al*. 2016). The cells were passaged with 1 mg/ml dispase (Roche, Mannheim, Germany) in DMEM with high glucose and a plastic scraper (Sumitomo Bakelite Co., LTD Tokyo, Japan). The cells were split at a ratio of 1:5–1:8 every 7 d.

### EB formation

EB formation was induced as described previously (Itskovitz-Eldor *et al*. 2000, International Stem Cell Initiative *et al*. 2007). Briefly, hPSCs were transferred using dispase (1 mg/ml; Roche) to plastic low-attachment dishes (at a 1:1 ratio) to allow the formation of their aggregations. Floating human EBs were maintained in DMEM with 10% FBS (EQITECH-BIO, Kerrville, TX) supplemented with 10 µM 2-mercaptoethanol or a serum-free differentiation medium, hESF-FX differentiation medium containing 10 µg/ml human insulin, 5 µg/ml apo-transferrin, 1 mg/ml rHSA, and 3F for 14 d. The culture medium of the hEBs was changed every 3 d.

### Early differentiation assay for growth factor response

Cells were seeded in 24-well or 96-well plates coated with 2 µg/cm^2^ fibronectin at a density of 10,000–20,000 cells/well. Cultures were maintained with hESF-FX medium for 1 d. The next day, the cells were stimulated with BMP4 (10 ng/ml) in the hESF-FX differentiation medium without insulin or ascorbic acid. For neuroectodermal differentiation, the cells were stimulated with 10 µM SB43542 (Wako) and 10 µM DMH1 (Wako) in StemFit AK02N medium (Ajinomoto, Tokyo, Japan). The culture medium was changed daily.

### Hematopoietic cell differentiation

Human iPS cells were dissociated into single cells with Accutase (Merck-Millipore, Burlington, MI). The cells were resuspended in differentiation medium I (mTeSR1 (STEMCELL Technologies, Vancouver, Canada), 50 μg/ml ascorbic acid, 0.45 mM MTG, 2 mM l-glutamine, and antibiotics) supplemented with 10 ng/ml human BMP4, 1 ng/ml Activin A, and 10 μM Y-27632 and were then plated on low-attachment dishes (day 0). On day 2, cell aggregates were cultured in differentiation medium II (Iscove’s modified Dulbecco’s medium (IMDM), 50 μg/ml ascorbic acid, 0.45 mM MTG, 2 mM L-glutamine, and antibiotics) supplemented with 10 ng/ml human BMP4 and 5 ng/ml human VEGF. On day 4, half of the medium was changed to differentiation medium II supplemented with 10 ng/mL human BMP4, 5 ng/mL human VEGF, and 10 μM SB431542. On day 6, cell aggregates were cultured in differentiation medium II supplemented with 2 ng/ml human BMP4, 5 ng/ml human VEGF, 10 ng/ml human stem cell factor (SCF) (Peprotech, Rocky Hill, NJ), and 10 ng/ml human thrombopoietin (TPO) (Peprotech). On day 10, differentiated cells were analyzed by flow cytometry with an LSR Fortessa flow cytometer.

### Hepatic cell differentiation

hPSCs were differentiated as discussed previously (Si-Tayeb *et al*. 2010; Mallanna and Duncan 2013; Yanagihara *et al*. 2016). Briefly, hPSCs were harvested using Accutase and plated at 600,000 cells per well in 24-well plates precoated with 300 µl/well Geltrex (Thermo Fisher Scientific, Waltham, MA). Approximately 24 h after seeding the cells with mTeSR1 (Stemcell Technologies), when the cells were 85–95% confluent, differentiation was initiated by culture for 5 d with 50 ng/ml Activin A (R&D Systems) in RPMI/B27 (without insulin) supplement (Invitrogen) under ambient oxygen/5% CO_2_. In addition, we included 10 ng/ml BMP4 (R&D Systems) and 20 ng/ml FGF2 (R&D Systems) for the first 2 d. Then, the cells were cultured for 5 d with 20 ng/ml BMP4 (R&D Systems)/10 ng/ml FGF-2 (R&D Systems) in RPMI/B27 (containing insulin) under 4% O_2_/5% CO_2_, then for 5 d with 20 ng/ml HGF (R&D Systems) in RPMI/B27 (containing insulin) under 4% O_2_/5% CO_2_, and finally for 5 d with 20 ng/ml Oncostatin-M (R&D Systems) in Hepatocyte Culture Media (Lonza) supplemented with SingleQuots (without EGF) in ambient oxygen/5% CO_2_.

### Immunocytochemistry

Immunocytochemistry was performed as described (Aihara *et al*. 2010; Hayashi *et al*. 2010, 2007). The image analysis was performed with In Cell analyzer 2000 and Developer toolbox software (GE Healthcare, Little Chalfont, Buckinghamshire, UK) or a confocal microscope LSM 880 (CarlZeiss, Oberkochen, Germany). Antibodies used in this study were anti-human OCT4 (H-134), rabbit polyclonal IgG (sc-9081, Santa Cruz Biotechnology, Santa Cruz, CA; dilution 1/500), and goat anti-rabbit IgG (H + L) conjugated with Alexa Fluor 546 (A11035, Life Technology; Dilution 1/2000).

### Flow cytometry

Cells were incubated with an APC-conjugated anti-human CD34 antibody (clone: 581, Biolegend San Diego, CA) and FITC-conjugated anti-human CD43 antibody (clone: 1G10, BD, Franklin Lakes, NJ) at 4°C for 30 min and washed twice with staining buffer (PBS/2% FBS). The analysis was performed on an LSR Fortessa flow cytometer using FACS Diva software (BD). The data were analyzed using FlowJo software (Tommy Digital Inc., Tokyo, Japan).

### Albumin secretion assay

The culture media were replaced with fresh media, and the conditioned media were harvested 24 h later at 20 d after hepatocyte differentiation. The human albumin content in the supernatant was determined using the Human Albumin ELISA Quantitation kit (Bethyl Laboratory, Montgomery, TX). Human serum albumin (HSA) (Bethyl Laboratory) was used as the standard, and the primary antibody (1:100; Bethyl Laboratory) and secondary antibody (1:150,000; Bethyl Laboratory) were used according to the manufacturer’s instructions. Albumin levels were normalized to the total RNA of the cells per well.

### RT-qPCR analysis

Total RNA was isolated from hPSCs or hPSC-derived cells using the RNeasy mini or micro kit (Qiagen, Valencia, CA) and treated with DNase I (Qiagen) to remove any genomic contamination. For customized quantitative PCR analysis, 500 ng or 1 µg of RNA was used for reverse transcription with the Superscript VILO cDNA synthesis kit (Thermo Fisher Scientific). For real-time PCR analysis, PCR was performed based on TaqMan or SYBR Green gene expression technology with a 7300 Real-Time PCR System (Thermo Fisher Scientific), following the manufacturer’s instructions. Threshold cycles were normalized to the housekeeping gene GAPDH and translated to relative values. The primer sequences for each primer set are shown in Table S3 in the supplementary material. For RT2 Profiler™ PCR, 500 ng of RNA was used for reverse transcription with an RT2 First Strand Kit (Qiagen). Total RNA was isolated from undifferentiated hiPSCs, differentiated hepatocytes, the HeppG2 cell line, fetal liver hepatocytes, and adult hepatocytes. Gene expression was analyzed using the RT2 Profiler™ PCR Array: custom PCR array (consisting of hepatocyte lineage-specific genes) (Qiagen) with RT2 SYBR Green/ROX qPCR Master Mix (Qiagen) and an ABI7900 HTR-PF (Applied Biosystems, Foster City, CA).

For the scorecard differentiation evaluation assay, 1 µg of RNA was used for reverse transcription with the TaqMan hPSC Scorecard Panel High Capacity cDNA RT kit with RNase Inhibitor (Thermo Fisher Scientific). The investigation of gene expression was carried out using the TaqMan hPSC Scorecard™ Panel (Thermo Fisher Scientific) with the TaqMan Gene Expression Master Mix (Thermo Fisher Scientific) and an ABI7900 HTR-PF (Thermo Fisher Scientific). Data analysis was carried out using the Web-based hPSC Scorecard Analysis Software at www.lifetechnologies.com/scorecarddata.

### Microarray analysis

Gene expression microarray experiments were performed by DNA Chip Research Inc (Tokyo, Japan). After obtaining the genomic DNA-free total RNA described above, the RNA quantity and quality were verified with a NanoDrop 1000 (Thermo Fisher Scientific), Qubit 2.0 Fluorometer (Thermo Fisher Scientific), and Bioanalyzer RNA6000 Nano (Agilent, Santa Clara, CA). After complementary RNA (cRNA) was synthesized, the cRNA was hybridized with SurePrint G3 Human GE microarray 8 × 60 K (Design ID: 39,494). To scan the microarray image, an Agilent C scanner (Agilent) was used. To quantify the fluorescence intensity, Feature Extraction 10.7.3 software (Agilent) was used. All the data were uploaded to GEO (Gene Expression Omnibus) as GSE120772.

The statistical test was performed using Welch’s *t*-test with unpaired, unequal variance. Each *p*-value was calculated asymptotically. To calculate the FDR, the Benjamini–Hochberg correction was applied. Clustering analysis was performed on all the microarray probes using “pvclust” function with method.hclust = “average,” method.dist = “correlation,” nboot = 100 in R program.

### Teratoma formation

The cells were harvested by dispase treatment, collected into tubes, and centrifuged, and the pellets were suspended in DMEM. A total of 1,000,000 hESCs were injected into the rear leg muscle or thigh muscle of a SCID (C.B-17/lcr-scid/scidJcl) mouse (CLEA Japan, Tokyo, Japan). Seven weeks after injection, tumors were dissected, weighed, and fixed with 10% formaldehyde Neutral Buffer Solution (Nacalai tesque, Kyoto, Japan). Paraffin-embedded tissue was sliced and stained with hematoxylin and eosin. All animal experiments were conducted in accordance with the guidelines for animal experiments of the National Institute of Biomedical Innovation, Health, and Nutrition, Osaka, Japan.

### Karyotype analysis

Log-phase hPSCs (days 3–4 of subculture) were treated with metaphase arresting solution (Genial Genetic Solutions Ltd., Cheshire, UK) and 0.02–0.04 μg/ml podophyllotoxin (Sigma-Aldrich) for 90–120 min before chromosome preparation. The number of chromosomes was counted in 20 metaphase cells using a Nikon ECLIPSE Ni microscope and NIS Elements Br Software (Nikon, Tokyo, Japan). mFISH staining was performed using a 24XCyte Human Multicolor FISH Probe kit (MetaSystems GmbH Altlussheim, Germany) and analyzed with a Zeiss Axio Imager up-light microscope and the Isis FISH Imaging System (Carl Zeiss).

### CGH array analysis

Genomic DNA was isolated from hESCs using the Allprep DNA/RNA micro kit (Qiagen). CGH analysis was outsourced to DNA Chip Research Inc.

### Statistical analysis

At least three independent experiments were performed. Statistical analysis of the data was performed with a *t*-test. *p* < 0.05 was considered significant. Values are reported as the mean ± SD.

## Results

### Characterization of trisomy 12 sublines of hPSCs

**Figure 1.**
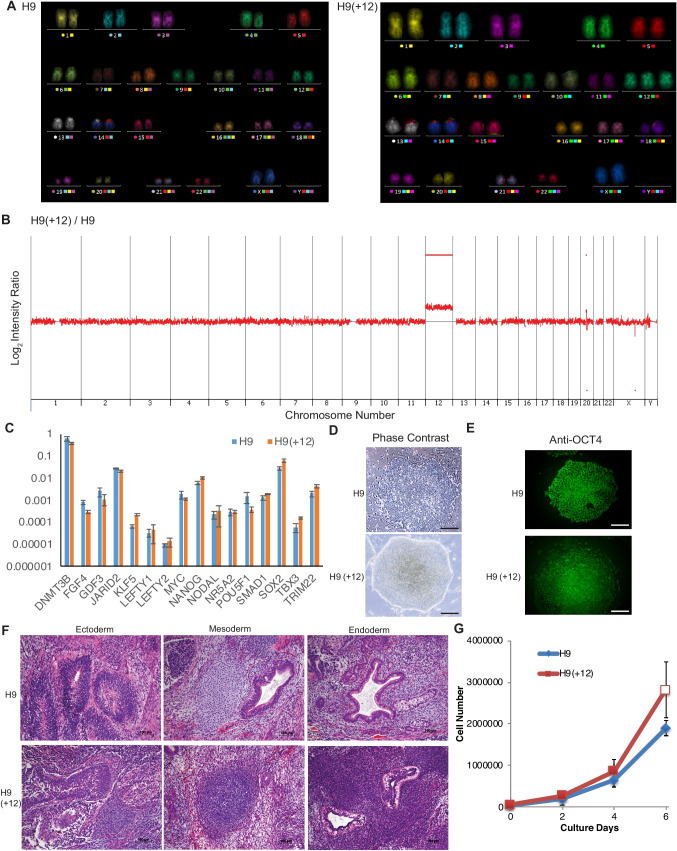
Characterization of the trisomy 12 hESC (H9) subline. (*A*) Whole chromosome painting FISH karyotyping of the H9 (*left panel*) and H9(+ 12) (*right panel*) lines. (*B*) CNV (copy number variations) of the H9(+ 12) from the CGH array analysis. (*C*) The panel of RT-qPCR data on the H9(+ 12) and H9 lines. Values are shown as the means ± SE (*n* = 3). (*D*) Phase contrast images of the H9 and H9(+ 12) lines. *Scale bars*, 100 µm. (*E*) Immunocytochemistry images of OCT4 protein in the H9 and H9(+ 12) lines. *Scale bars*, 100 µm. (*F*) Teratoma sections of the H9 and H9(+ 12) lines generated in NOD-SCID mice. Representative areas of ectodermal, mesodermal, and endodermal tissues are shown. *Scale bars*, 100 µm. (*G*) The growth curves of H9 and H9(+ 12) lines. Values are shown as the means ± SE (*n* = 3).

After prolonged culture, we identified a subline of H9 hESCs that carried trisomy 12, designated H9(+ 12). G-band karyotyping analysis detected trisomy 12 in all of the cells analyzed in the H9(+ 12) subline at passage 65 (Figure S1A; right panel). Whole-chromosome painting FISH (fluorescence in situ hybridization) analysis confirmed the presence of trisomy 12 in all of the cells analyzed in the H9(+ 12) subline (Fig. 1*A*; right panel) at passage 81. CGH (comparative genomic hybridization) array analysis revealed that the entire chromosome 12 region was amplified in the H9(+ 12) subline (Fig. 1*B*). STR (short tandem repeat)-PCR analysis confirmed that these cell lines originated from the same donor (data not shown). RT-qPCR, phase contrast imaging, and immunocytochemistry of OCT4 protein confirmed that the H9(+ 12) subline maintained its PSC morphology and self-renewal marker expression pattern (Fig. 1*C*–*E*). Teratomas generated from these sublines contained three germ layer derivatives (Fig. 1*F*). These results indicate that the H9(+ 12) subline stably carries an extra copy of whole chromosome 12 while maintaining its self-renewal capacity and pluripotency. The PDTs (population doubling times) of H9 and H9(+ 12) lines were 32.7 h and 27.8 h, respectively, which were calculated from growth curves (Fig. 1*G*). Notably, we observed that trisomy 12 increased the cell proliferation rate in concordance with a previous report (Ben-David *et al*. 2014).

We also identified a subline of 201B7 hiPSCs, designated 201B7-1A, that carried an extra copy of chromosome 12 after prolonged culture and showed pluripotency and self-renewal (Kato *et al.* 2016). We further characterized this subline using CGH array analysis and revealed that the entire chromosome 12 region was amplified in the 201B7-1A subline (Figure S1B). These results indicated that the 201B7-1A subline stably carried an extra copy of whole chromosome 12. We also identified a subline of 19–9-7 T hiPSCs, designated 19–9-7 T(+ 12), which carried an extra copy of chromosome 12 (Figures S2A, B) after prolonged culture and showed pluripotency and self-renewal (Figures S2C-F). We further characterized this subline using CGH array analysis and revealed that the entire chromosome 12 region was amplified in the 19–9-7 T subline (Figure S2G). STR-PCR analysis confirmed that these cell lines originated from the same donor (data not shown). These results indicated that the 19–9-7 T subline stably carried an extra copy of whole chromosome 12.

### Trisomy 12 sublines show specific gene expression changes

**Figure 2.**
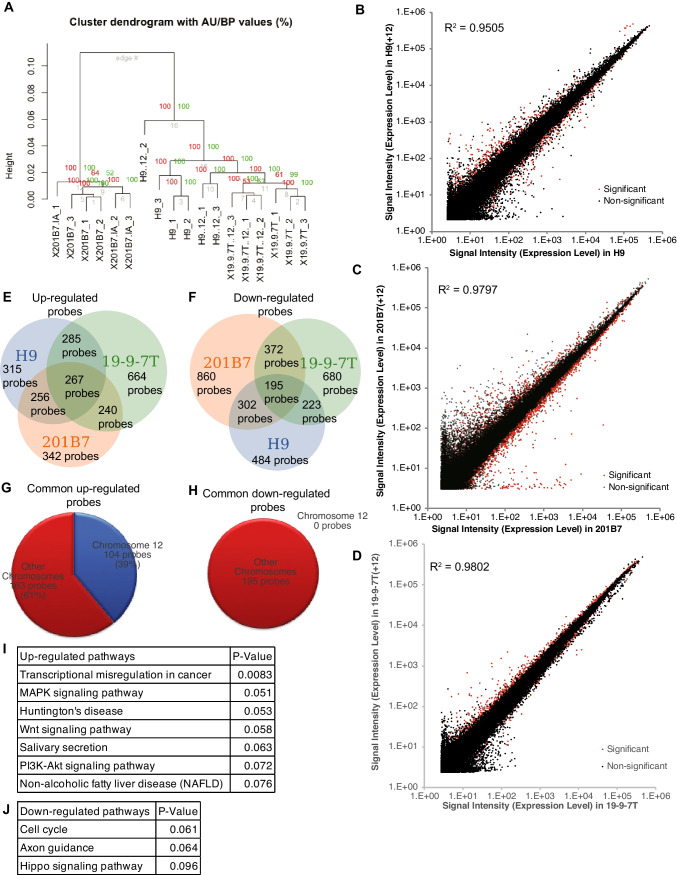
Comparison of global gene expression in trisomy 12 hPSC lines. (*A*) Cluster dendrogram of microarray data from trisomy 12 hPSC lines and their original lines. *N* = 3 from each lines. (*B–D*) Scatter plot of signal intensity for all microarray probes. Each *dot* in the plot shows the mean signal intensity of each probe averaged from 3 samples of H9 (*X-axis*) and H9(+ 12) (*Y-axis*) hESC lines (*B*), 201B7 (*X-axis*) and 201B7(+ 12) (*Y-axis*) hiPSC lines (*C*), and 19–9-7 T (*X-axis*) and 19–9-7 T(+ 12) (*Y-axis*) hiPSC lines (*D*). (*E*, *F*) *Pie charts* of significantly upregulated (*E*) or downregulated (*F*) probes of the trisomy 12 hPSC lines in common from microarray analysis (FDR < 0.1). (*G*, *H*) *Pie* charts of significantly upregulated *G* or downregulated *H* probes of the trisomy 12 hPSC lines in common from microarray analysis (FDR < 0.1). The area in *blue* indicates the ratio of the probes targeting chromosome 12. The area in *red* indicates the probes targeting the other chromosomes. (*I*, *J*) The list of “PANTHER” pathways and their *p* values extracted from the commonly upregulated genes *I* and downregulated gene *J.*

We examined global gene expression in the trisomy 12 hPSC sublines compared with the parental lines using microarray analysis. We performed microarray on biological triplicate samples of H9, H9(+ 12), 201B7, 201B7-1A, 19–9-7 T, and 19–9-7 T(+ 12) PSC lines. First, unsupervised hierarchical clustering analysis on all the samples was performed. In the cluster dendrogram (Fig. 2*A*), the samples from the same line were situated in quite similar positions, validating the sample data quality of each cell line. Also, the samples from the trisomy 12 sublines were positioned close to the original normal PSC lines, rather than to the other trisomy lines. These results suggested that the trisomy 12 sublines largely remained the characteristics of original cell lines in their global gene expression patterns. The averaged expression levels of all probes from H9 line/H9(+ 12) subline, 201B7 line/201B7-1A subline, and 19–9-7 T line/19–9-7 T(+ 12) subline were plotted, respectively (Fig. 2*B*–*D*). The correlation coefficients between the H9 line and the H9(+ 12) subline, 201B7 line and 201B7-1A subline, and 19–9-7 T line and 19–9-7 T(+ 12) subline were 0.9505, 0.9797, or 0.9802, respectively. These results indicated that these cell lines showed similarities in their overall gene expression patterns that were quite high. Differentially regulated probes between original PSC lines and trisomy 12 sublines in common with all the 3 lines were extracted (Fig. 2*E* and *F*). Focusing on probes targeting the genes in chromosome 12, 104 probes were significantly upregulated (*p* < 0.05) in H9(+ 12), comprising 39% of all the upregulated probes (Fig. 2*G*). In contrast, none of the probes targeting chromosome 12 was downregulated (Fig. 2*H*). Considering that chromosome 12 contains approximately 1000 genes (~ 5% of the whole genes) (from HUGO Gene Nomenclature Committee; https://www.genenames.org/cgi-bin/statistics?c=12), these results may rightly reflect the effects of the entire trisomy 12 on the global expression profiles of the same original hiPSC line. Next, we extracted common upregulated and downregulated probes among these three trisomy 12 PSC lines compared to their original PSC lines (Tables S1 and S2, respectively) to examine gene characteristics by pathway analysis. From the commonly upregulated probes, transcriptional misregulation in cancer, MAPK signaling pathway, Huntington’s disease, Wnt signaling pathway, salivary secretion, PI3K-Akt signaling pathway, and non-alcoholic fatty liver disease (NAFLD) were significantly overrepresented (Fig. 2*I*). From the commonly downregulated probes, cell cycle, axon guidance, and Hippo signaling pathway were significantly overrepresented (Fig. 2*J*). These results suggest that trisomy 12 affects gene expression in these specific signaling pathways in addition to cancer-related cell cycle pathways. Also, because these signaling pathways are critical for the self-renewal and differentiation of PSCs, we hypothesized that trisomy 12 affected the developmental potentials and/or propensities regulated by these signaling pathways.

### Trisomy 12 sublines exhibit compromised mesendodermal differentiation propensity in embryoid bodies cultured in serum-free medium

**Figure 3.**
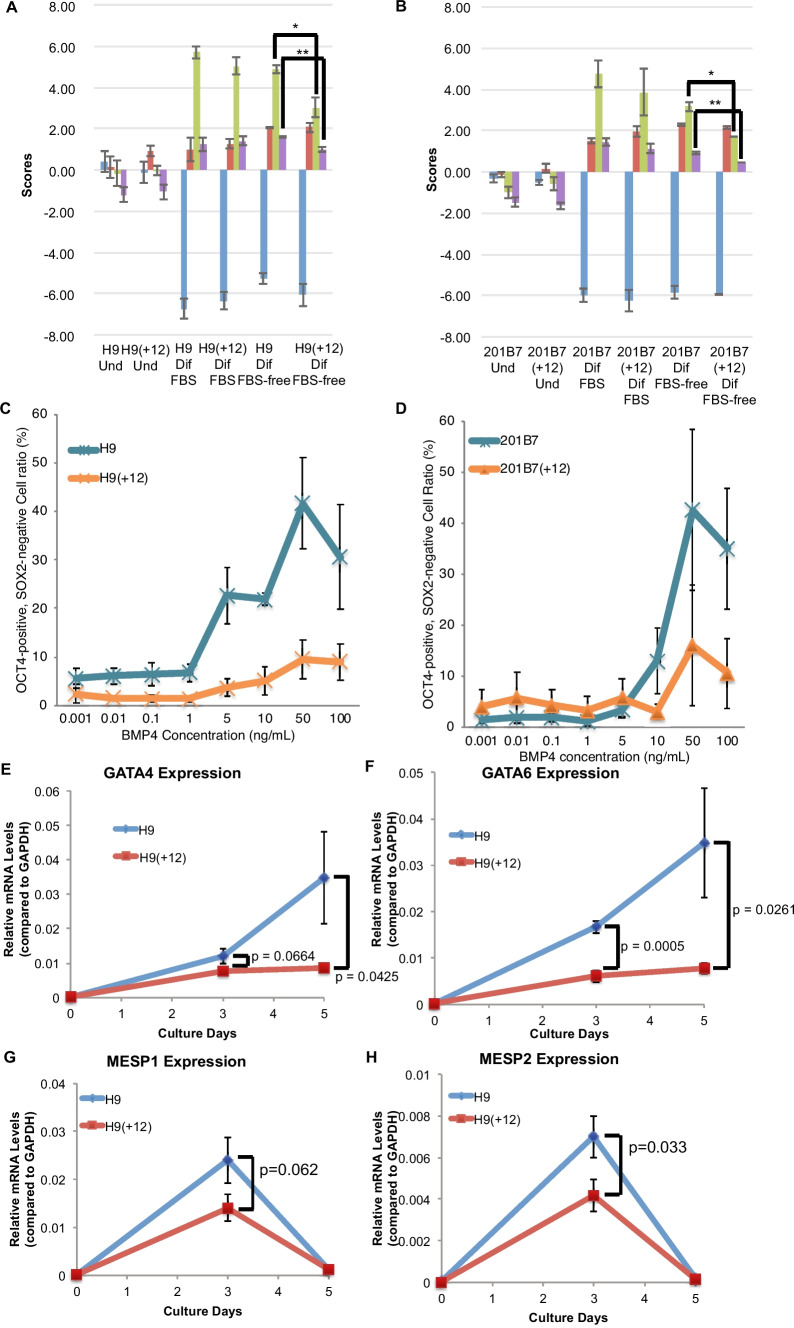
Trisomy 12 hPSC sublines exhibit compromised differentiation of a primitive streak in the response to BMP4. (*A*, *B*) Scorecard analysis was performed on undifferentiated or hPSC-derived EBs in the H9 or H9(+ 12) hESC lines *A* and the 201B7 or 201B7(+ 12) hiPSC lines *B*. Values are shown as the means ± SE (*n* = 3). *p < 0.05 and **p < 0.01 from *t*-tests. (*C*, *D*) The ratio of cells that exited from a self-renewal status toward the mesendodermal lineage (OCT-4-positive and SOX2-negative) in the response to different BMP4 concentrations for 2 d. The H9 or H9(+ 12) hESC lines *C* and the 201B7 or 201B7(+ 12) hiPSC lines *D* were shown. Values are shown as the means ± SE (*n* = 3). (*E*–*H*) The expression levels of *GATA4 E*, *GATA6 F*, *MESP1 G*, and *MESP2 H* in BMP4-containing serum-free medium for 0, 3, or 5 d were detected with RT-qPCR. Values are shown as the means ± SE (*n* = 3). *p* values were calculated from *t*-tests.

To examine the differentiation propensities in the trisomy 12 PSC lines, we spontaneously differentiated these hPSC lines into three germ layers in vitro. EBs (embryoid bodies) were formed in either the serum-containing medium or serum-free medium. We assumed that differentiating cells might exhibit their differentiation propensity more clearly in the less-nutrient serum-free medium, by relying on cell-intrinsic signals. After collecting the differentiated cells in the EBs, gene expression patterns were examined using TaqMan hPSC Scorecard Panel assays (Bock *et al*. 2011). In the serum-containing medium, the EBs from the H9(+ 12) subline showed similar expression patterns in three germ layers to those of the EBs from the H9 parental lines (Fig. 3*A*). However, in the serum-free medium, the EBs from the H9(+ 12) subline exhibited impaired expression patterns of mesodermal and endodermal markers compared with the EBs from the H9 parental lines, but the expression patterns of self-renewal or ectodermal genes were not changed between the H9(+ 12) and H9 lines. These trends were also observed in the 201B7-1A subline compared with the original 201B7 hiPSC line (Fig. 3*B*). Because there were no exogenous factors for inducing mesendodermal lineages in the serum-free medium, these results indicated that these trisomy 12 sublines carried impaired cell-autonomous differentiation propensity toward the mesodermal and endodermal lineages, although their overall differentiation potentials toward these lineages were not impaired.

### Trisomy 12 sublines exhibit compromised mesendodermal differentiation induced by BMP4

We examined the effects of specific inducers of mesendodermal differentiation in trisomy 12 hPSC sublines in a serum-free medium. BMP4 is a potent mesendodermal inducer from pluripotent stem cells, which is conserved in vertebrates (Hogan 1996, Niehrs *et al*. 2000, Faial *et al*. 2015). Undifferentiated hPSCs were treated with different concentrations of BMP4 for 2 d, then OCT4-positive and SOX2-negative cells, which represent an exit from self-renewal toward mesendodermal lineages (Wang *et al*. 2012), were examined. As the concentration of BMP4 increased, the ratio of OCT4-positive and SOX2-negative cells increased; however, the rate of the increase was smaller in H9(+ 12) than in H9 (Fig. 3*C*). Similarly, 201B7(+ 12) exhibited a lower ratio of OCT4-positive and SOX2-negative cells in response to BMP4 treatment (Fig. 3*D*). These results indicated that the trisomy 12 hPSC sublines exhibited poor differentiation responses with BMP4 treatment.

The expression levels of early mesendodermal marker genes were examined in H9 and H9(+ 12) cells in response to induction with 10 ng/ml BMP4. The expression levels of key transcription factors, namely, *GATA4*, *GATA6*, *MESP1*, and *MESP2*, were increased by BMP4; however, the rate of upregulation was significantly suppressed in the H9(+ 12) subline (Fig. 3*E*–*H*). Other mesendodermal transcription factors, namely, T and Hand1, were similarly upregulated in both the H9 and H9(+ 12) lines (Figures S3A and B). Additionally, the expression levels of *SOX7*, an extraembryonic endodermal marker (Seguin *et al*. 2008), and *NANOG*, a self-renewal marker (Chambers *et al*. 2003; Mitsui *et al*. 2003), were similarly downregulated in both the H9 and H9(+ 12) lines (Figures S3C and D). These results indicated that the gene expression of specific critical factors in mesendodermal differentiation was dysregulated in BMP4-treated trisomy 12 hPSC lines, although these cells could properly respond to the BMP4 signal.

We also tried to compare the differentiation efficiency toward early ectodermal lineages. We employed dual SMAD inhibition methods to differentiate into neuroepithelial cells, which are marked by PAX6 expression. We found that the differentiation efficiency of PAX6-positive neuroepithelial cells in trisomy 12 sublines was comparable to that in the original PSC lines (Figures S4A-F).

### Direct differentiation into hematopoietic cell lineages is compromised in trisomy 12 sublines

**Figure 4.**
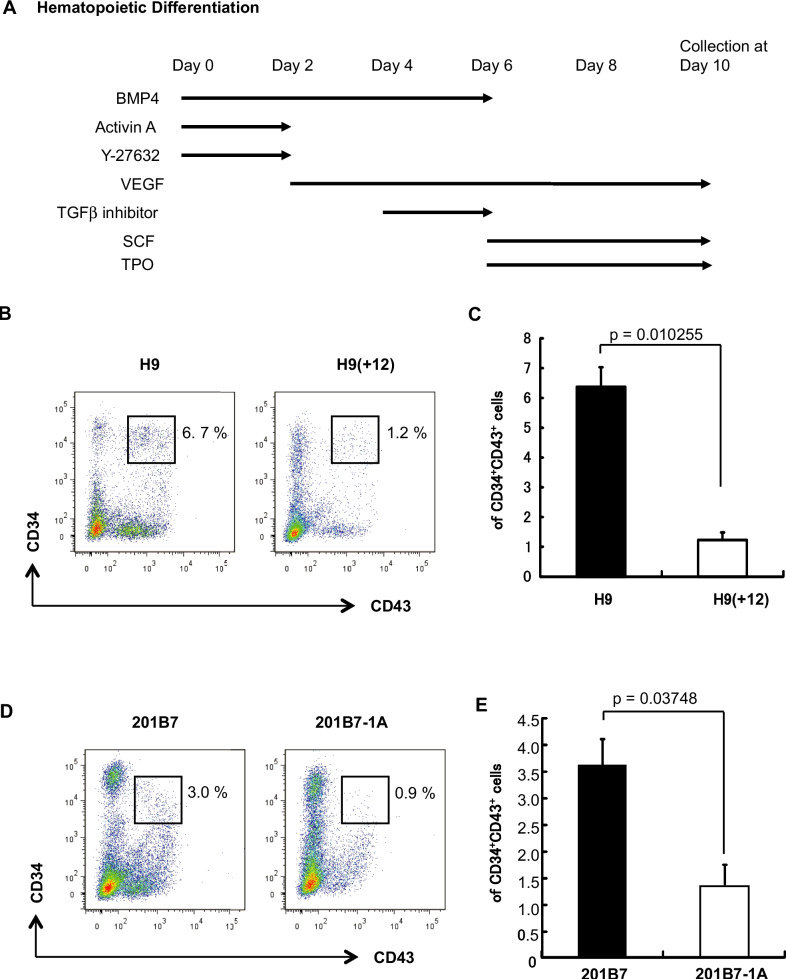
The hematopoietic differentiation propensity of hPSCs is compromised by trisomy 12. (*A*) Schemes of direct differentiation into hematopoietic lineages by hPSCs. (*B*) Representative flow cytometry data showing the ratio of CD43-CD34-double-positive hematopoietic lineage cells from the H9 or H9(+ 12) lines. (*C*) The percentages of double-positive cells are shown. Values are shown as the means ± SE (*n* = 3). *p* values were calculated with *t*-tests. (*D*) Representative flow cytometry data showing the ratio of CD43-CD34-double-positive hematopoietic lineage cells from the 201B7 or 201B7-1A lines. (*E*) The percentages of double-positive cells are shown. Values are shown as the means ± SE (*n* = 3). *p* values were calculated from *t*-tests.

We examined differences between the parental and trisomy 12 hPSCs in terms of the direct differentiation of mesendodermal derivatives. First, we examined direct differentiation into hematopoietic lineages using reported methods (Fig. 4*A*). Ten days after differentiation, CD34 and CD43 double-positive hematopoietic progenitor cells (Vodyanik *et al*. 2006) were analyzed by flow cytometry. We found that the ratio of these double-positive cells was lower in the H9(+ 12) subline than in the parent H9 line (Fig. 4*B* and *C*). Additionally, the ratio of these double-positive cells was decreased in the 201B7-1A subline compared with the parent 201B7 line (Fig. 4*D* and *E*). These results indicated that trisomy 12 comprised hematopoietic differentiation in the direct differentiation analysis in vitro.

### Hepatic differentiation is compromised in trisomy 12 sublines

**Figure 5.**
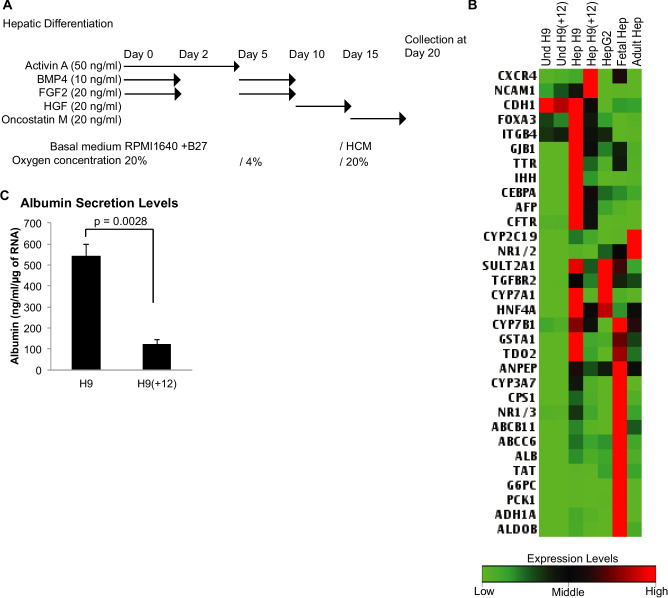
Impaired hepatic differentiation from H9(+ 12) cells. (*A*) Schemes of direct differentiation into hepatocytes from hPSCs. (*B*) Heatmaps of the expression levels of hepatocyte marker genes were determined by custom RT-PCR array. Gene expression significantly differed between H9(+ 12) and H9 in triplicate experiments (*p* < 0.05). (*C*) Albumin secretion from differentiated cells from H9 and H9(+ 12) lines. The albumin levels in the supernatant were measured with ELISA and normalized to the total RNA of the cells in each well. Values are shown as the means ± SE. *p* values were calculated from *t*-tests (*n* = 3).

We examined direct hepatic differentiation using previously established methods (Si-Tayeb *et al*. 2010; Mallanna and Duncan 2013) (Fig. 5*A*). Twenty days after differentiation, gene expression profiles were examined using a hepatocyte qPCR panel. The gene expression patterns, which were significantly different between differentiated H9(+12) and H9, are shown in a heat map (Fig. 5*B*). While the expression of many hepatocyte-specific genes was upregulated in the differentiated hepatocyte-like cells from the H9 hESC line, the expression levels of most upregulated genes were lower in the H9(+12) subline (Fig. 5*B*). Albumin secretion levels were also examined in these conditions using ELISA. The albumin secretion levels were lower in H9(+12) than in the parental H9 line (Fig. 5*C*). These results indicated that trisomy 12 compromised the hepatic differentiation propensity in the direct differentiation analysis in vitro.

## Discussion

In this study, we first demonstrated that trisomy 12 compromises the mesendodermal differentiation propensities of hPSCs. hPSC sublines carrying trisomy 12 that were identified from three established hPSC lines showed lower propensities toward mesendodermal differentiation in EBs cultured in the serum-free medium. Exit from the self-renewal state induced by BMP4 was impaired in the trisomy 12 hPSC sublines, with less upregulation of key transcription factors. Direct differentiation of these trisomy 12 hPSC sublines into hematopoietic or hepatic lineages was also impaired. The analysis of the global gene expression profiles implied that the expression patterns of genes involved in key cell signaling pathways that were required for proper mesendodermal differentiation might be altered in the trisomy 12 hPSCs.

The effect of trisomy 12 on the cell cycle has been examined because hPSCs carrying trisomy 12 have a strong selection advantage in hPSC culture conditions (Catalina *et al*. 2008; Mayshar *et al*. 2010). Our trisomy 12 hPSC lines showed an accelerated cell proliferation rate. Previous reports have suggested that trisomy 12 confers a proliferation advantage in hPSC cultures mainly by increasing cell replication in culture, and trisomy 12 hPSCs are more sensitive to several cytotoxic replication inhibitors, further demonstrating their increased proliferation (Ben-David *et al*. 2014). This replication advantage is particular to undifferentiated hPSCs because the proportions of replicating cells in neural differentiation cultures are similar between neural cells derived from diploid hPSCs and those derived from trisomy 12 hPSCs (Ben-David *et al*. 2014). Another study reported negative selection against trisomy 12 in teratomas transplanted from mixtures of normal and trisomy 12 hESCs (Gertow *et al*. 2007). These findings suggest that the compromised mesendodermal differentiation propensities of the hPSCs might not be due to the increased proliferation rate of the trisomy 12 hPSC lines in their undifferentiated state.

We demonstrate that the expression patterns of genes involved in the Wnt signaling pathway are dysregulated in the trisomy 12 sublines compared with the parental lines from transcriptome analysis. Wnt signaling pathways have pleiotropic roles in the proliferation, self-renewal, and early differentiation of hPSCs, with conflicting reports claiming that Wnt signaling promotes either self-renewal or differentiation (Sato *et al*. 2004; Nakanishi *et al*. 2009; Davidson *et al*. 2012; Kurek *et al*. 2015; Xu *et al*. 2016). Thus, predicting the outcomes of hPSC behavioral changes based on changes in the gene expression patterns of Wnt signaling components in trisomy 12 hPSCs is difficult. Combined with functional differentiation assays, gene expression assays showed that changes in overall gene expression directed trisomy 12 hPSCs to suppress differentiation toward the mesendodermal lineages induced by BMP4. Several studies have shown that crosstalk occurs between BMP signaling and cadherin/Wnt signaling as a downstream molecular mechanism of mesendodermal development (reviewed in Walsh *et al*. 2010). One study used *Xenopus* embryos to show that Smad1 and β-catenin activated by the combinatorial Wnt and BMP signaling pathways co-occupy hundreds of cis-regulatory DNA elements and negatively regulate BMP ligand expression in the foregut (Stevens et al. 2017). A future study involving the dissection of the molecular mechanisms and identification of the critical regulators in trisomy 12 hPSCs will be interesting.

## Conclusions

We have revealed the critical consequences of trisomy 12 on the differentiation propensities of hPSCs. Because detecting trisomy 12 in routine culture experiments other than karyotyping and detailed staining or sequencing methods is difficult, the use of hPSC lines containing trisomy 12 cells unknowingly leads to critical errors in assays addressing differentiation and other processes. Previous reports have shown potential tumorigenicity in hPSC-derived cell transplantation because trisomy 12 is also the most common chromosomal abnormality in various germ cell tumors (Samaniego *et al*. 1990; Riopel *et al*. 1998). Transplanted endothelial cells that are differentiated from trisomy 12 human embryonic stem cells cause tumor-like tissue formation (Moon *et al*. 2011). Additionally, trisomy 12 increases the tumorigenicity of hPSCs *in vivo*, inducing transcriptionally distinct teratomas from which pluripotent cells can be recovered (Ben-David *et al*. 2014). These studies provide warnings against the use of hPSCs carrying trisomy 12 in regenerative medicine. In addition to these previous studies, our findings warn against the use of hPSCs carrying trisomy 12 for *in vitro* studies in terms of basic sciences and drug development and pinpoint the importance of routine examinations of genomic integrity to support correct and reproducible results. Although our use of only three lines in this study might not be enough to draw these conclusions, our detailed assays on specific growth factors in serum-free medium combined with statistical tests should be valuable for evaluating differentiation propensities based on cell-intrinsic properties.

### Supplementary Information

Below is the link to the electronic supplementary material.

Supplementary file1 (PDF 1177 KB)

Supplementary file2 (XLSX 89 KB)

Supplementary file3 (XLSX 95 KB)

## Data Availability

Microarray data were openly available in GEO (Gene Expression Omnibus) as GSE120772. Other data that support the findings of this study are available on request from the corresponding authors.
