# Supplementary material for: Trisomy 12 compromises the mesendodermal differentiation propensity of human pluripotent stem cells
Source: In Vitro Cell Dev Biol Anim. 2024 Jan 2;60(5):521–34. doi: 10.1007/s11626-023-00824-9 (PMC11126453; doi:10.1007/s11626-023-00824-9)
Supplement: Supplementary file 1 — Supplementary file1 (PDF 1177 KB) [file 11626_2023_824_MOESM1_ESM.pdf]

Supplementary Information

Trisomy 12 compromises the mesendodermal differentiation propensity of human pluripotent stem cells

Kana Yanagihara, Yohei Hayashi, Yujung Liu, Tomoko Yamaguchi, Yasuko Hemmi, Minako Kokunugi, Mari Wakabayashi, Kozue Uchio Yamada, Ken Fukumoto, Mika Suga, Satoshi Terada, Hiroki Nikawa, Kenji Kawabata, Miho Furue

Supplementary Figure S1 - S4

Supplementary Table 1 and 2: spreadsheet files

Suppmentary Table 3

Supplementary Figures

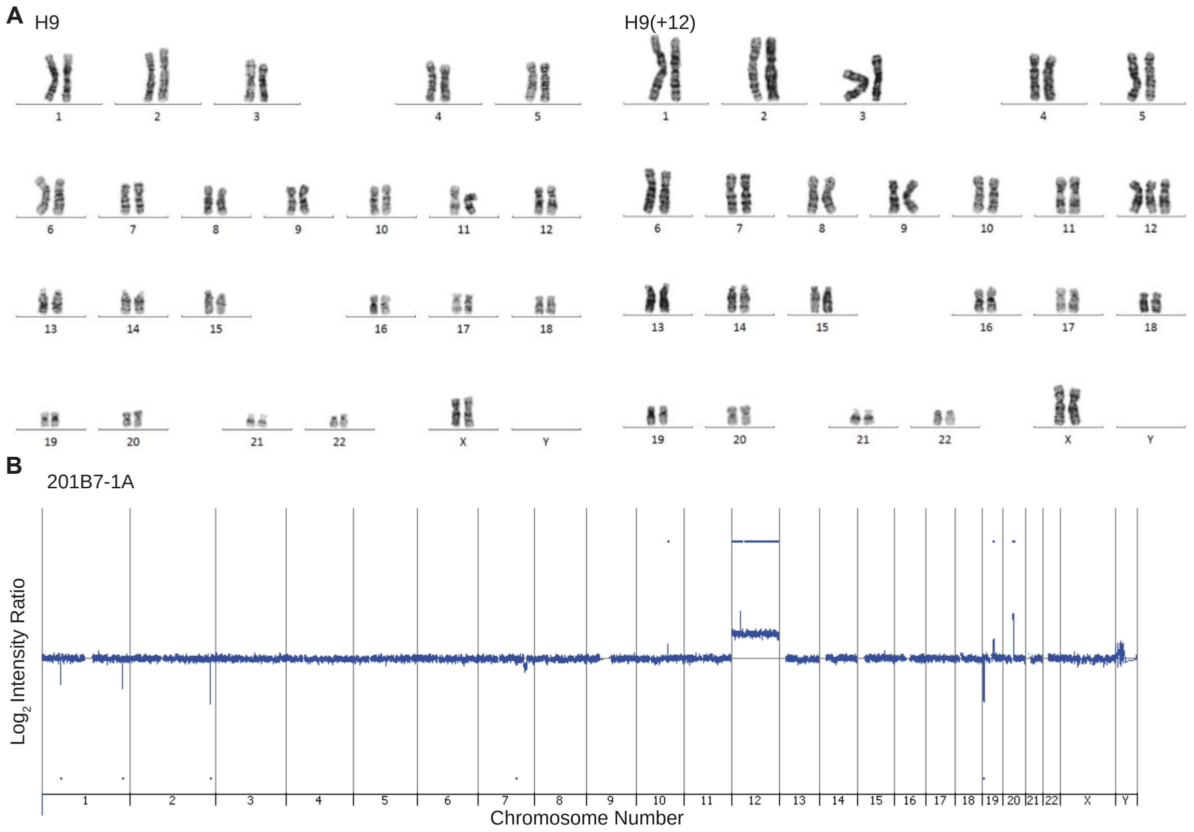

Figure S1. Characterization of the trisomy 12 hPSC sublines (continued from Figure 1.

(A) G-band karyotyping of the H9 (left panel) and H9(+12) (right panel) lines. (B) CNVs in the 201B7-1A detected with CGH array analysis.

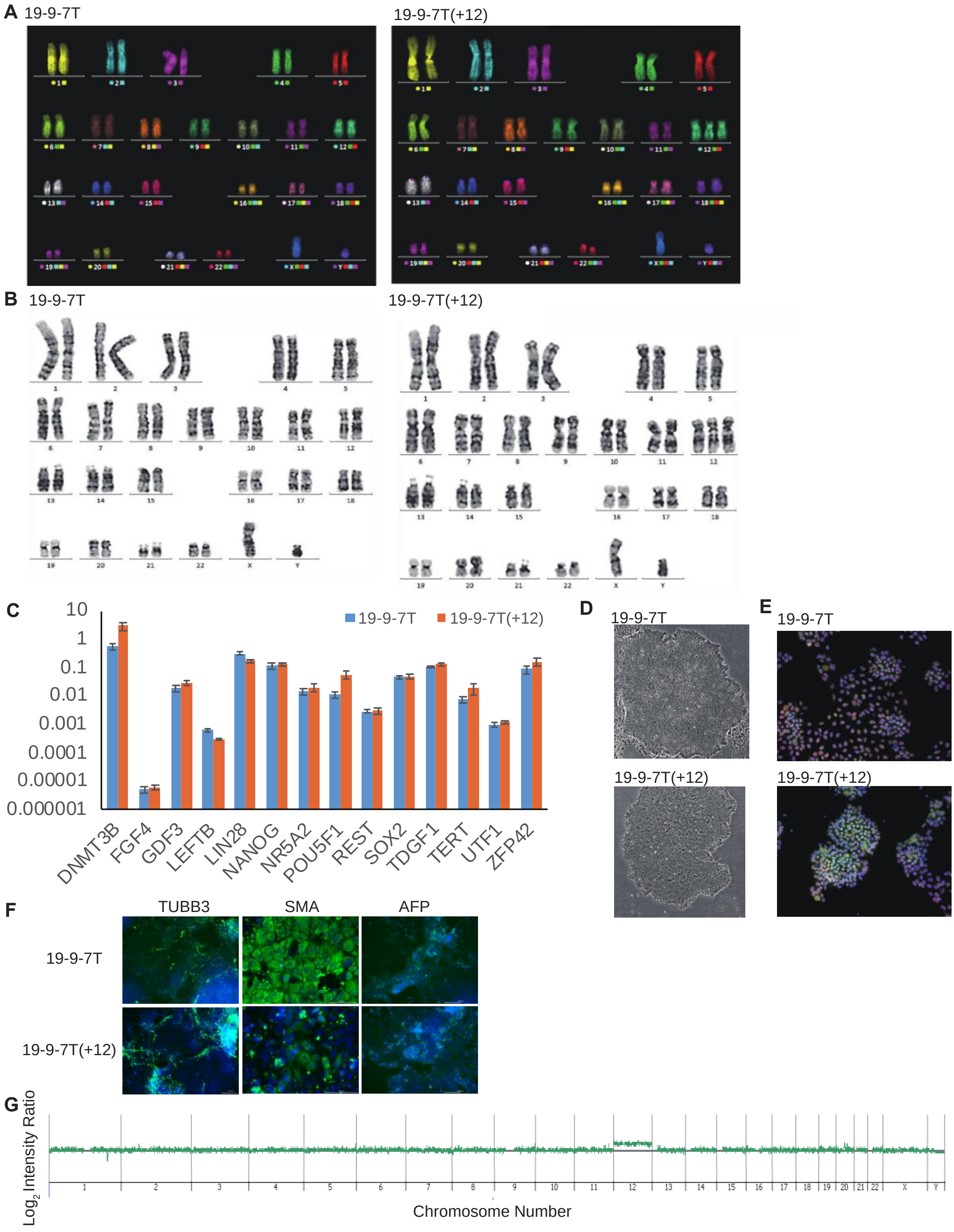

Figure S2. Characterization of the trisomy 12 19-9-7T hiPSC subline (continued from Figure 1).

(A) Whole chromosome painting FISH karyotyping of the 19-9-7T (left panel) and 19-

9-7T(+12) (right panel) lines. (B) G-band karyotyping of the 19-9-7T (left panel) and 19-9-7T(+12) (right panel) lines. (C) The panel of RT-qPCR data on the 19-9-7T(+12) and 19-9-7T lines. Values are shown as the means +/- SE (n=3). (D) Phase contrast images of the 19-9-7T(+12) and 19-9-7T lines. (E) Immunocytochemistry image of NANOG (Green) and OCT4 (Red) in the 19-9-7T and 19-9-7T(+12) subline. Nuclei is counterstained with DAPI (Blue). (F) Immunocytochemistry image of differentiation markers, TUBB3, SMA, and AFP (Green) in the differentiated embryoid bodies (EBs) from 19-9-7T and 19-9-7T(+12) lines. (G) CNVs in the 19-9-7T(+12) subline from the CGH array analysis.

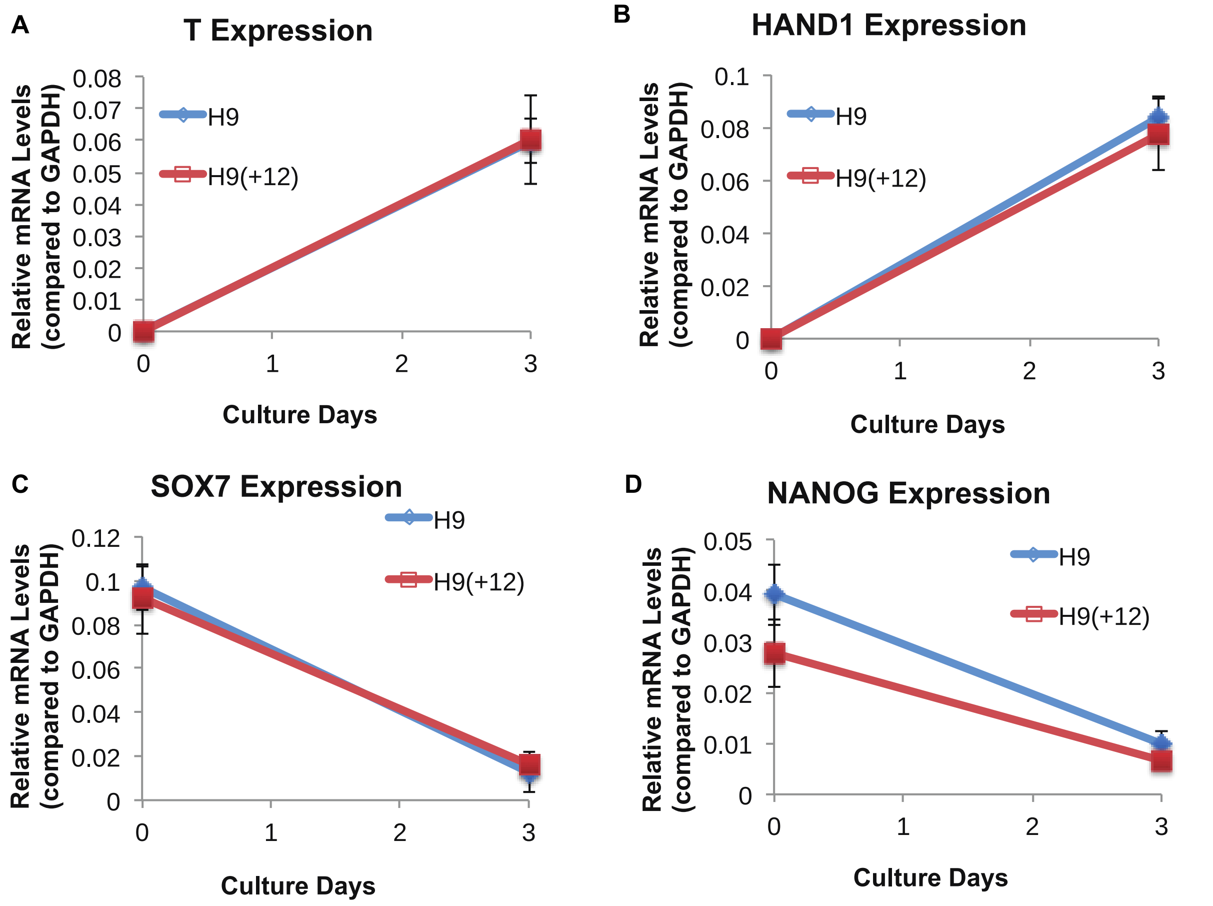

Figure S3. H9 (+12) cells exhibit compromised differentiation of a primitive streak in response to BMP4 (continued from Figure 3).

(A-D) The expression levels of *T* (A), *HAND1* (B), *SOX7* (C), and *NANOG* (D) in H9 and H9(+12) lines in a BMP4-containing serum-free medium for 0 or 3 days were detected by RT-qPCR. Values are shown as the means +/- SE (n=3).

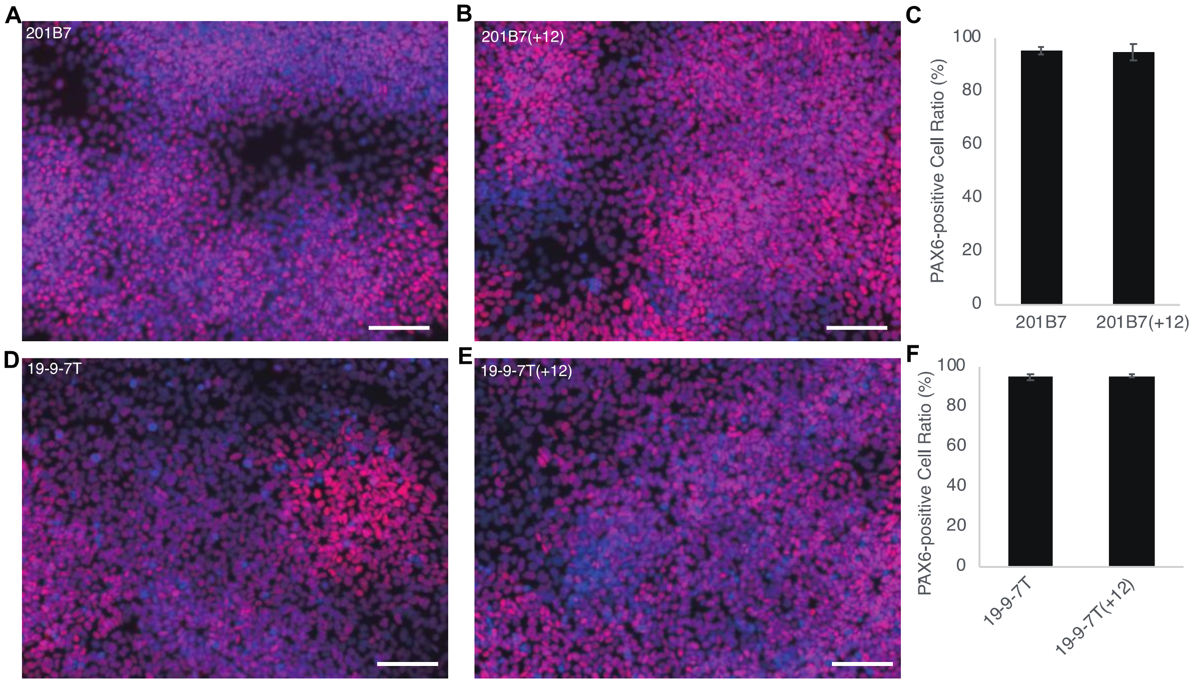

Figure S4. Trisomy 12 hPSC sublines showed normal differentiation potentials to neurectoderm (continued from Figure 3).

(A, B) Representative images of immunocytochemistry of PAX6 (Red) in 201B7 (A) and 201B7(+12) (B) after 5 days of differentiation culture for neurectodermal induction conditions. Nuclei were counterstained with DAPI (Blue). Scale bars, 100 µm. (C) The ratio of PAX6-positive cells calculated from the data of immunocytochemistry. (D, E) Representative images of immunocytochemistry of PAX6 (Red) in 19-9-7T (D) and 19-9-7T(+12) (E) after 5 days of differentiation culture for neurectodermal induction conditions. Nuclei were counterstained with DAPI (Blue). Scale bars, 100 µm. (F) The ratio of PAX6-positive cells calculated from the data of immunocytochemistry.

Table S1. Commonly upregulated probes among trisomy 12 hPSC sublines (spreadsheet)

Table S2. Commonly downregulated probes among trisomy 12 hPSC sublines (spreadsheet)

Table S3. List of primers used in this study

Gene Name	Forward	Reverse
GATA4	TCCAAACCAGAAAACGGAAGC	GCCCGTAGTGAGATGACAGG
GATA6	GCGGGCTCTACAGCAAGATG	ACAGTTGGCACAGGACAATCC
MESP1	GAAGTGGTTCCTTGGCAGAC	TCCTGCTTGCCTCAAAGTGT
MESP2	GCAGTGTACCAGGGTCTCTCT	ACTGTGGCTCCAGCACCT
SOX17	CAGAATCCAGACCTGCACAA	CTCTGCCTCCTCCACGAA
FOXA2	GGGAGCGGTGAAGATGGA	TCATGTTGCTCACGGAGGAGTA
T	TGCTTCCCTGAGACCCAGTT	GATCACTTCTTTCCTTTGCATCAAG
HAND1	GTG AGA GCA AGC GGA AAA G	GTG CGT CCT TTA ATC CTC TTC
SOX7	ACGCCGAGCTCAGCAAGAT	TCCACGTACGGCCTCTTCTG
NANOG	TGAACCTCAGCTACAAACAG	TGGTGGTAGGAAGAGTAAAG
GAPDH	CAAAGTTGTCATGGATGACC	CCATGGAGAAGGCTGGGG
